# Evaluation of circulating tumor DNA as a prognostic and predictive biomarker in *BRAF* V600E mutated colorectal cancer—results from the FIRE‐4.5 study

**DOI:** 10.1002/1878-0261.13778

**Published:** 2024-12-04

**Authors:** Susanne Klein‐Scory, Alexander Baraniskin, Wolff Schmiegel, Thomas Mika, Roland Schroers, Swantje Held, Kathrin Heinrich, David Tougeron, Dominik P. Modest, Ingo Schwaner, Jan Eucker, Rudolf Pihusch, Martina Stauch, Florian Kaiser, Christoph Kahl, Meinolf Karthaus, Christian Müller, Christof Burkart, Sebastian Stintzing, Volker Heinemann

**Affiliations:** ^1^ Department of Internal Medicine, Universitaetsklinikum Knappschaftskrankenhaus Bochum GmbH Ruhr University Bochum Germany; ^2^ Department of Hematology, Oncology and Palliative Care Evangelisches Krankenhaus Hamm gGmbH Germany; ^3^ ClinAssess GmbH Leverkusen Germany; ^4^ Department of Oncology LMU University Hospital Munich Germany; ^5^ Department of Hepato‐Gastroenterology Poitiers University Hospital and University of Poitiers France; ^6^ Department of Hematology, Oncology, and Cancer Immunology (CCM) Charité—Universitaetsmedizin Berlin Germany; ^7^ Onkologische Schwerpunktpraxis Kurfürstendamm Berlin Germany; ^8^ Department of Hematology, Oncology, and Cancer Immunology (CBF) Charité—Universitaetsmedizin Berlin Germany; ^9^ MVZ Praxis Pihusch Rosenheim Germany; ^10^ Hematology, Oncology/Hemostaseology Kronach Germany; ^11^ VK&K Studien GmbH Landshut Germany; ^12^ Klinikum Magdeburg gGmbH, Department of Hematology Oncology and Palliative Care Magdeburg Germany; ^13^ Department of Internal Medicine, Clinic III – Hematology, Oncology and Palliative Care Rostock University Medical Center Germany; ^14^ Department of Hematology, Oncology and Palliative Care München Klinik Harlaching and Neuperlach Germany; ^15^ Evang. Kliniken Essen‐Mitte Germany; ^16^ Schwarzwald‐Baar Klinikum Villingen‐Schwenningen Germany; ^17^ German Cancer Consortium (DKTK) German Cancer Research Centre (DKFZ), Site Berlin Heidelberg Germany; ^18^ Department of Medicine III, LMU Klinikum Comprehensive Cancer Center Munich Germany; ^19^ German Cancer Consortium (DKTK) German Cancer Research Centre (DKFZ), Site Munich Heidelberg Germany

**Keywords:** bevacizumab, cetuximab, circulating tumor DNA, FOLFOXIRI, liquid biopsy, mutational load

## Abstract

The randomized FIRE‐4.5 (AIO KRK0116) trial compared first‐line therapy with FOLFOXIRI (folinic acid, fluorouracil, oxaliplatin, and irinotecan) plus either cetuximab or bevacizumab in B‐Raf proto‐oncogene, serine/threonine kinase (*BRAF*) V600E‐mutant metastatic colorectal cancer (mCRC) patients. This study was accompanied by a prospective translational project analyzing cell‐free circulating tumor DNA (ctDNA) in plasma to test whether ctDNA analysis may help to guide clinical treatment decision making. FIRE‐4.5 included mCRC patients with *BRAF* V600E mutation detected by tissue‐based analyses. Liquid biopsies (LBs) were collected at baseline (pre‐treatment) and during therapy. Digital droplet PCR (ddPCR) technology was applied for determination of *BRAF* mutations and the *in vitro* diagnostics (IVD)‐certified ONCOBEAM *RAS* procedure for analysis of *RAS* mutations. The *BRAF* V600E variants in ctDNA were analyzable in 66 patients at start of the therapy, at baseline. No *BRAF* V600E mutations were detected in 26% (17/66) of patients and was associated with a significantly longer progression‐free survival (PFS: 13.2 vs 6.5 months; HR 0.47; *P =* 0.014) and overall survival (OS: 36.8 vs 13.2 months; HR 0.35; *P =* 0.02) as compared to ctDNA mutant patients. Patients with detectable *BRAF* mutations showed a clear superiority of FOLFOXIRI plus bevacizumab with regard to PFS (10.4 vs 5.7 months; HR 0.4; *P =* 0.009) and OS (16.6 vs 11.6 months; HR 0.5; *P =* 0.15), while this was not the case for *BRAF* wild‐type patients. Follow‐up LBs were obtained from 51 patients. Patients converting from *BRAF* V600E mutant to a *BRAF* V600 wild‐type status (36%, *N* = 18) had a superior PFS (8.6 vs 2.3 months; *P =* 0.0002) and OS (17.4 vs 5.1 months; *P* < 0.0001) compared to patients with stable or increased mutational allele frequency (12%, *N* = 6). Those patients also achieved a significantly greater disease control rate (89% vs 20%; *P =* 0.008). In conclusion, LB evaluating ctDNA is informative and may help to guide treatment in patients with *BRAF* V600E‐mutated mCRC.

## Graphical abstract

Liquid biopsy evaluating circulating tumor DNA as a prognostic and predictive biomarker is informative and may help to guide treatment in patients with *BRAF* V600E mutated colorectal cancer. In patients with a detectable *BRAF* V600E variant in ctDNA, chemotherapy with bevacizumab showed superiority in terms of PFS and OS, while this was not the case in *BRAF* wild‐type patients.

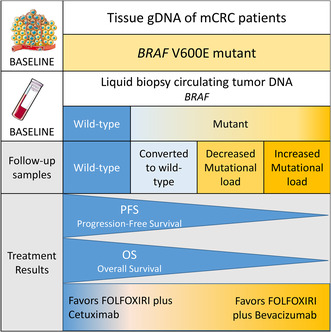

**Abbreviations**
BEV: bevacizumab treatment in combination with FOLFOXIRI
BL: baseline liquid biopsy
CET: cetuximab treatment in combination with FOLFOXIRI
CI: confidence interval
ctDNA: circulating tumor DNA
DCR: disease control rate
FOLFOXIRI: treatment combination with 5‐Fluorouracil‐Folinic acid‐Oxaliplatin‐Irinotecan
HR: hazard ratio
MAF: mutational allele frequency %
mCRC: metastasized colorectal cancer
*N*
: number of patients
OR: odds ratio
ORR: objective response rate
OS: overall survival
PD: progression disease
PFS: progression‐free survival

## 1 Introduction

Colorectal cancer (CRC) accounts for approximately 6–10% of all malignancies and is one of the leading causes of cancer‐related deaths worldwide [1]. Molecular subgroups with heterogeneous genetic patterns define prognosis and treatment possibilities. Therefore, upfront molecular testing before start of first‐line treatment is recommended by international guidelines [2, 3, 4, 5, 6]. These tests include mutational analyses in hot spot regions of in Kirsten rat sarcoma virus (*KRAS*), *neuroblastoma ras* viral oncogene homolog (*NRAS*), and the *b‐raf* proto‐oncogene (*BRAF*), since only tumors without activating mutations in the epidermal growth factor receptor (EGFR) pathway benefit from treatment with anti‐EGFR antibodies [7, 8]. In addition, defective DNA mismatch repair (dMMR) or microsatellite instability (MSI‐h) have been established to predict efficacy of immune‐checkpoint inhibitors [9]. In more than 50% of mCRC patients, *RAS* mutations are detected in the tumor tissue. Additionally, about 10% of CRC tumors harbor a *BRAF* V600E somatic mutation in the *BRAF* gene being mutually exclusive with *RAS* mutations [10, 11].

FIRE‐4.5 is the first prospective and randomized study investigating the efficacy of intensive chemotherapy with FOLFOXIRI (5‐fluorouracil, folinic acid, oxaliplatin, irinotecan) combined with either cetuximab, an anti‐EGFR monoclonal antibody, or bevacizumab, an anti‐VEGF monoclonal antibody, in the first‐line treatment of *BRAF* V600E‐mutant mCRC. Outcome data have previously been published showing superior ORR and significantly longer PFS for FOLFOXIRI plus bevacizumab and no significant difference in OS between both treatment arms [12].

Monitoring of ctDNA during treatment of mCRC patients revealed that the clonal composition of the tumor appears to be rearranged in response to treatment [13, 14, 15, 16]. Recent studies demonstrated that clonal evolution is induced by selective pressure of therapeutic agents and may lead to changes in *RAS* mutation status. The reduction of *RAS* mutational load and the conversion to a *RAS* wild‐type state is associated with better clinical outcomes [17, 18, 19, 20]. Analogous to *RAS* mutations, also *BRAF* mutations can be determined by analysis of ctDNA [21]. The measurement of ctDNA has not been established for detection of early‐stage CRC in asymptomatic patients, yet [22, 23].

Here, we report results of a translational project along with FIRE‐4.5 trial that monitored ctDNA for *RAS*‐ and *BRAF* V600E mutations. To detect possible changes in mutational allele frequency, blood samples were collected prior to therapy onset (at baseline) and during treatment. ctDNA from plasma was analyzed for *BRAF* mutations using digital droplet polymerase chain reaction (ddPCR), while *RAS* mutations were detected with the IVD certified OncoBEAM technology [24, 25].

## 2 Materials and methods

### 2.1 Patients and ethical statement

FIRE‐4.5 (AIO KRK0116, NCT04034459) was a randomized study that included mCRC patients with proven *BRAF* V600E mutation based on tissue analyses. According to a 2 : 1 randomization, patients were distributed to arm A (*N* = 72), where they received FOLFOXIRI plus cetuximab or arm B (*N* = 35), where FOLFOXIRI plus bevacizumab was applied. The trial was in compliance with the Declaration of Helsinki and was registered with EudraCT no. 2015‐004849‐11. The protocol was approved by the responsible ethics committees of the participating centers. Patients provided written informed consent before trial entry. Details of the study have previously been reported [26].

Tissue analyses (including gene mutation analyses) were performed de‐centrally within the routine diagnostic process in different pathology departments. A prospectively planned translational program was associated to the FIRE‐4.5 trial to investigate the clinical relevance of liquid biopsies. The liquid biopsy samples were collected in 59 participating centers and transferred overnight to the Universitätsklinikum Knappschaftskrankenhaus Bochum GmbH, Ruhr University Bochum, Germany. First liquid biopsy sample was taken in September 2017 and the last sample in October 2021. Blood samples were obtained in cell‐free DNA (cfDNA) collection tubes (Cell‐Free DNA BCT; Streck™, La Vista, Nebraska, USA) according to the trial design of the FIRE‐4.5 study. Blood samples were taken before treatment onset (baseline samples BL) and thereafter at predefined intervals after start of therapy, and then at the timepoint of first event (Table S1, Fig. S1). Briefly, the baseline liquid biopsy samples were taken pre‐treatment (median 0 days, IQR (interquartile range) −7 to −2), the follow‐up samples 1 (FU) were taken after 60 days (IQR 56–69), the follow‐up 2 after 175 days (IQR 132–211), and finally, the follow‐up 3 after 315 days (IQR 275–420).

All cfDNA isolation and analyses were performed at the Universitätsklinikum Knappschaftskrankenhaus Bochum GmbH, IMBL, Ruhr University Bochum, Germany. Patients´ characteristics are summarized in Table S2. In contrast to routine procedure of tissue testing for *RAS* and *BRAF* mutations, the analyses presented here were carried out centrally and using the same method.

### 2.2 cfDNA isolation from plasma and detection of *RAS* and *BRAF* V600E variants

Plasma isolation was done 3 days after blood collection at the latest. Briefly, blood cells were removed by centrifugation at 1600 ***g*** for 10 min, and further cleared by a second centrifugation at 6000 ***g*** for 10 min as previously described [27]. Plasma was carefully collected and stored at −80°C until further use. cfDNA was extracted using a QIAamp circulating nucleic acid kit (Qiagen™ #55114, Hilden, Germany), used according to the manufactures' instructions. The plasma volume was 3 mL and the elution volume was 140 μL according to manufacturer's protocol and [28]. The median cfDNA amount of plasma samples was 17.6 ng·mL^−1^ (95%CI 14.9–22.05 ng·mL^−1^) calculated by ddPCR. cfDNA amount per mL was calculated with sum of copies·μL^−1^ * volume of assay/volume input * total volume of eluate/ volume * 3.3/10^3^ of plasma according to [29]. The results are calculated in copies·μL^−1^ based on a Poisson distribution curve using quantasoft software (version 1.7.4.0917, Bio‐Rad Laboratories GmbH, Feldkirchen, Germany).

*RAS* mutational status was analyzed by the IVD certified ONCOBEAM *RAS* procedure (Sysmex Corp., Kobe, Japan) that tests 34 allele variants of *KRAS‐* and *NRAS*‐genes with a cut‐off limit of 0.02% mutational allele frequency (MAF) as previously described [30].

ddPCR to detect *BRAF* V600E mutations was performed as previously described [25, 31]. Because of the high interlaboratory reproducibility and accuracy, the ddPCR‐based detection of *BRAF* V600E mutations can serve as a primary reference measurement procedure [25, 32]. ddPCR was performed using validated *BRAF* assays for mutant and wild‐type allele (*BRAF* mutant p.V600E c.1799T‐A #10031246, *BRAF* WT for p.V600E c.1799T>A #10031249 assay) in the QX200 workflow (Bio‐Rad Laboratories, Hercules, CA, USA) as previously described [33]. The median input amount for ddPCR analysis was 3.8 (95% CI 3.2–4.68). *BRAF* analyses were done at least in duplicate and at least four times for wild‐type results and low ctDNA amount. Only PCR reactions with more than 13 000 droplets were accepted.

The cut‐off level of the *BRAF* V600E variant analyses by ddPCR was calculated to 0.17% MAF (95%CI 0.15–0.19) with more than two mutant events considering detection limits and false positive results. Reproducibility and precision were assessed by replicate tests with reference material from Horizon discovery (Cambridge, UK) and additionally tests of cellular DNA obtained from gDNA of HT‐29 cell line (carrying *BRAF* V600E mutation) fragmented and spiked in wild‐type background DNA from Caco‐2 cell line (Fig. S2). Cell lines (HT‐29/CVCL_0320, Caco‐2/CVCL_0025) originated from ATCC were authenticated by STR (short tandem repeat) profiles according to CLS, DSMZ and ATCC databases and negatively tested for mycoplasma contamination by PCR. Linearity of the *BRAF* V600E assay was confirmed with titration of positive control sample in a wild‐type background. The mutation detection retains its linearity even at low total amounts of up to 2.5 ng total DNA in the assay.

For ddPCR, the whole amount of circulating fragmented DNA in the plasma samples was calculated by the sum of reference and mutant copies measured in respect to the reference samples in each experiment. It was thus possible to compare the whole amount and the MAF proportion of ctDNA, because the absolute numbers of copies were given and no preamplifications were done [34].

### 2.3 Statistical analysis

All statistical analyses and data plots were carried out using graphpad prism software (version 10.2.3, GraphPad Software Inc., Boston, Massachusetts, USA). Disease control rates (DCR: CR complete response + PR partial response + SD Stable disease) and objective response rate (ORR) as assessed by recist 1.1 guidelines (https://recist.eortc.org/recist‐1‐1‐2/). Comparison of patient rates (DCR and ORR) was performed with Fisher's exact test. Overall survival (OS) and progression‐free survival (PFS) were analyzed with Kaplan–Meier method and compared with log‐rank test. All tests were performed 2‐sided at significance level of 0.05. Hazard ratio (HR) and Odds ratio (OR) were given with the 95% confidence interval (95%CI). Data cut‐off for survival information in this analysis was January 2022.

## 3 Results

### 3.1 Patients characteristics

**Fig. 1:**
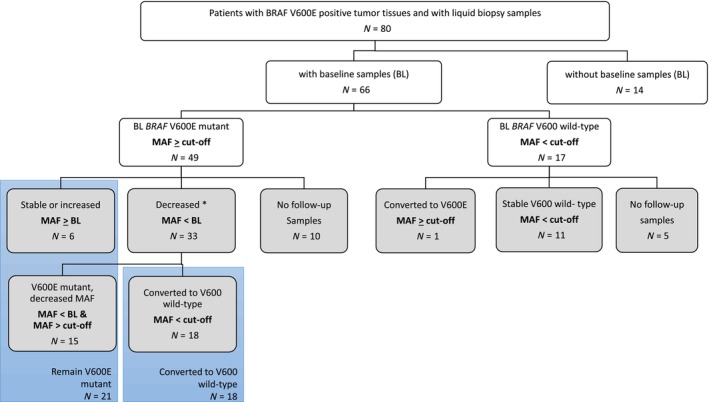
Consort diagram of patients with liquid biopsy subgrouped by mutational allele frequency of *BRAF* V600E in circulating tumor DNA (ctDNA). White boxes: Baseline liquid biopsy before treatment onset, gray boxes: Follow‐up sample collection during treatment, MAF‐mutational allele frequency (%), cut‐off value of the *BRAF* V600E MAF was set at 0.17 (see Fig. S2); BL baseline sample; * group of 7 patients with increased MAF in later follow‐up samples were included.

Overall, 107 patients with *BRAF* V600E mutated mCRC (based on analyses of tumor tissue) were included into the full analysis set of the FIRE‐4.5 study. In 80 patients, 161 liquid biopsy specimens were collected, whereby baseline specimens (BL, pre‐therapy) were obtained from 66 patients and 95 follow‐up specimens (FU) from 51 patients (Fig. 1, Tables S1 and S2).

### 3.2 Liquid biopsy results of baseline samples

In 74% of liquid biopsies collected from 66 patients at baseline (BL, pre‐therapy), the expected *BRAF* V600E mutation was found, while no *BRAF* mutation was observed in 26% (Fig. 1, Fig. S3). The amount of cell‐free DNA was not different in both groups (*P =* 0.753, *T*‐test with Welch's correction; Fig. S3). One BL sample was classified as primarily *RAS* mutated and was excluded from the study.

**Fig. 2:**
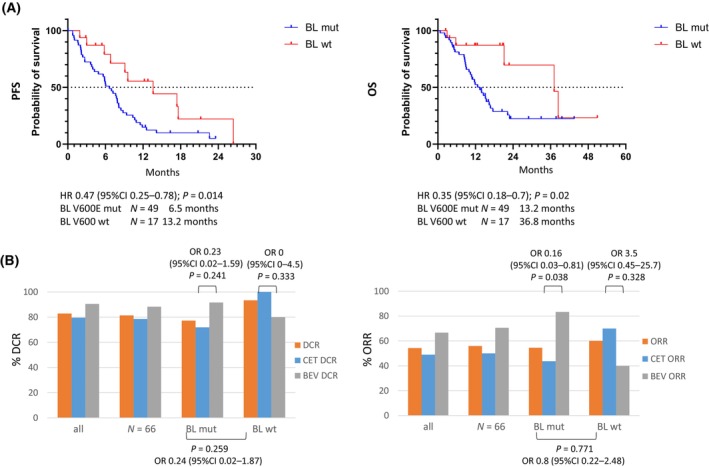
*BRAF* V600E status in ctDNA at baseline and patient outcome. (A) Survival curves of patients with BL containing only *BRAF* V600 wild‐type molecules versus patients with BL containing *BRAF* V600E mutant (BL mut) variants reveal the better outcome of wild‐type BL patients (BL wt). (B) Disease control rate (DCR) and objective response rate (ORR) for patients grouped by ctDNA *BRAF* mutational status of baseline samples (BL). ctDNA, circulating tumor DNA; CET, FOLFOXIRI plus cetuximab; BEV, FOLFOXIRI plus bevacizumab; PFS, progression‐free survival; OS, overall survival; HR, Hazard ratio with 95% confidence interval (95%CI) was calculated with Kaplan–Meier method, *P*‐value by log‐rank test; OR, Odds ratio calculated by Fisher's exact test. Dotted line indicates 50% probability of survival.

Regardless of the mutation status in tissue samples, BL *BRAF* V600 wild‐type status was associated with significantly better PFS (13.2 months versus 6.5 months; HR 0.47; *P =* 0.014) and OS (36.8 months versus 13.2 months; HR 0.35; *P =* 0.02) compared to BL *BRAF* V600E mutant patients (Fig. 2A). Also, the objective response rate [ORR: 60% (9 of 15) versus 55% (24 of 44)] and the disease control rate [DCR: 93% (14 of 15) versus 77% (34 of 44)] tended to be more favorable in patients with BL *BRAF* wild‐type (Fig. 2B).

### 3.3 Pattern of metastasis affects mutation detection rate

The discordancy between tissue and liquid biopsy *BRAF* V600E mutational status raised the question of whether the results of liquid biopsies depend on the primary tumor's location (sidedness) or on the pattern of metastasis. No difference in the BL mutational status was found regarding the sidedness of the primary tumor (Fig. S4A). However, the present study indicates that the findings obtained by liquid biopsy may relate to the pattern of metastasis (Fig. S4B). BL *BRAF* V600E mutations were more likely to be detected if metastases occurred only in the liver (OR 15.87; *P <* 0.0001) or in more than one organ including the liver (OR 16.12; *P <* 0.0001), or if lymph node metastases were present (OR 6.11; *P =* 0.018). By contrast, the detection of *BRAF* V600E variant at baseline (BL *BRAF* wild‐type) was less likely with peritoneal metastasis. Interestingly, the results of ctDNA status (*BRAF* V600E mut vs *BRAF* V600 wt) affected the outcome parameters, while there were no differences in outcome when the patients are stratified by site of metastasis (Fig. S5).

### 3.4 ctDNA analyses and treatment efficiency

**Fig. 3:**
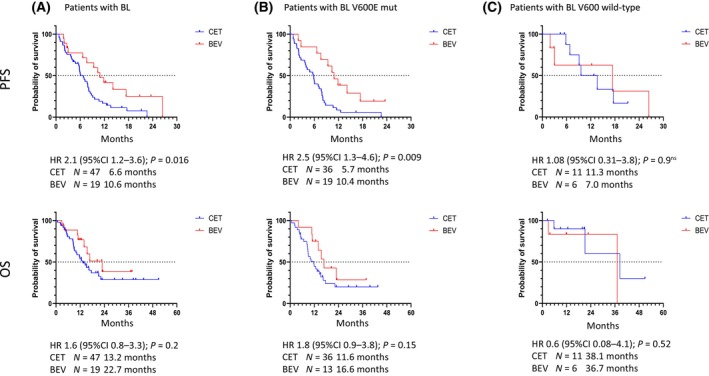
Comparison of treatment efficiency in patient groups classified by baseline liquid biopsy results. Survival curves of patients with liquid biopsy samples (A) with BL mut *N* = 49 (B) versus BL wt *N* = 17 (C). In the PFS time, a significant difference between the treatment arms could be measured: bevacizumab treatment seemed more beneficial for BL mutant group. In contrast, the efficiencies of both treatments were almost the same in the BL wild‐type patient group. BL, Baseline liquid biopsy before treatment onset; CET, FOLFOXIRI plus cetuximab; BEV, FOLFOXIRI plus bevacizumab; PFS, progression‐free survival; OS, overall survival; HR, Hazard ratio with 95% confidence interval (95%CI) and *P* values were calculated with Kaplan–Meier method and log‐rank test; Dotted line indicates 50% probability of survival.

Evaluation of results according to treatment arm confirmed that patients with BL *BRAF* V600E mutation showed inferior outcome if treated with cetuximab compared to bevacizumab (Fig. 3). They had a median PFS of 5.7 months versus 10.4 months (HR 2.5; *P =* 0.009) and a median OS of 11.6 months versus 16.6 months (HR 1.8; *P =* 0.15). Further, ORR [44% (14 of 32) versus 83% (10 of 12); *P =* 0.038] appears to be inferior in BL *BRAF* mutant patients and DCR tend to be worse [72% (23 of 32) versus 92% (11 of 12); *P =* 0.241] as well (Fig. 2B). In contrast, BL *BRAF* V600 wild‐type patients treated with cetuximab versus bevacizumab showed comparable survival parameters (Fig. 3C), while ORR [70% (7 of 10) versus 40% (2 of 5), *P =* 0.328] and DCR [100% (10 of 10) versus 80% (4 of 5); *P =* 0.333] appeared to favor the cetuximab arm (Fig. 2B).

### 3.5 Change of mutational status during treatment

Monitoring of the mutational allele frequency (MAF) during treatment may reflect the restructuring and clonal evolution of tumor cells [35, 36]. We examined the follow‐up samples of patients with BL *BRAF* mutant status (*N* = 39) and compared the MAF of the *BRAF* V600E mutation by ddPCR (Fig. S1). MAF deviations greater than 30% of the BL value were considered as relevant changes (Fig. S1C,D). The fold change was determined using the first available follow‐up value (MAF_FU_/MAF_BL_). The limit of 0.3 deviation should ensure that measurement outliers are not taken into account as a relevant change. In 6 patients (15%), MAF of the follow‐up samples had increased or was stable, whereby it is striking that all patients in this group were treated with cetuximab.

MAF decreased in 33 of 39 patients (84.6%) with *BRAF* V600E mutation at baseline. Of these, 18 patients converted to a *BRAF* V600 wild‐type status (Fig. 1 and Table S2). Patients in this group were approximately distributed 2 : 1 in the treatment groups, corresponding to the randomization pattern of the study. Among patients with decreased MAF, 6 patients (15.4%) later showed an increase of MAF (Fig. S1). Of these, 5 patients were treated in the cetuximab arm, in which no clinical progress was recorded within the observation period.

**Fig. 4:**
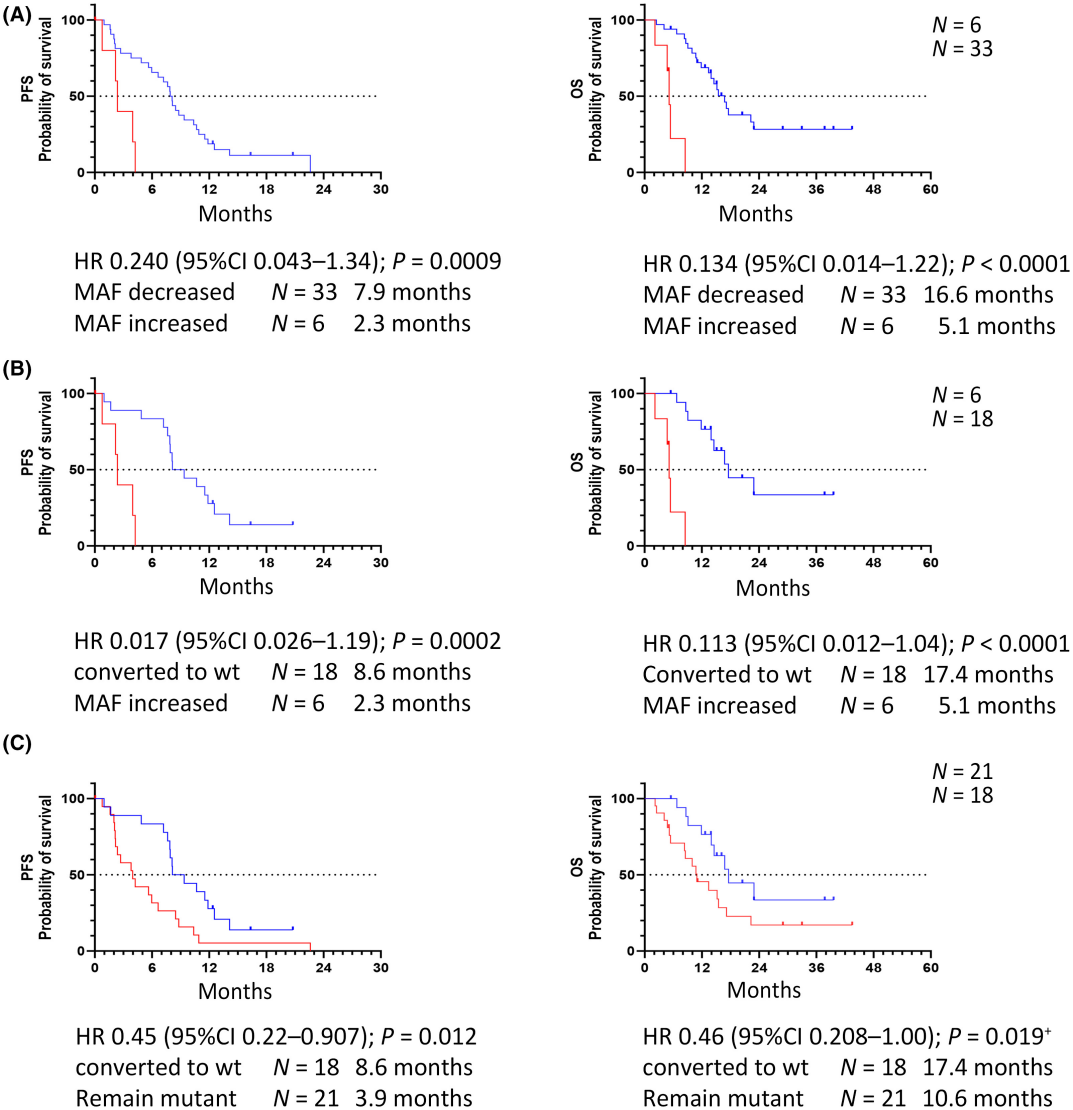
Grouping patients in respect to changes of mutational allele frequency (MAF %) in follow‐up liquid biopsies. The patients with initial *BRAF* V600E mutant ctDNA status and with increased MAF had the worst PFS and OS (A,B). On the other hand, the reduction in MAF is an indicator of good PFS and OS in response to treatment (C). OS, overall survival; PFS, progression‐free survival; *P*‐value were calculated by log‐rank test, except^+^. ^+^ *P*‐value was additionally calculated by Gehan‐Breslow‐Wilcoxon test, log‐rank *P =* 0.053 for this comparison. Dotted line indicates 50% probability of survival.

Patients with a decrease of MAF (*N* = 33) had a superior PFS (7.9 months vs 2.3 months, HR 0.24; *P =* 0.0009) and OS (16.6 months vs 5.1 months HR 0.13; *P <* 0.0001) than patients with stable or increased MAF (*N* = 6) (Fig. 4). Likewise, also DCR [81% (26 of 32) versus 20% (1 of 5); *P =* 0.013, OR 17.3 95%CI 1.94–217] and ORR [59% (19 of 32) versus 20% (1 of 5); *P =* 0.159, OR 5.84 (95%CI 0.75–74.6)] were markedly superior in the responding group (Table S2, Fig. S6). Comparable data of treatment arms were obtained for patients that converted to a *BRAF* wild‐type status (*N* = 18).

Patients (*N* = 6) in the cetuximab arm with an increased MAF showed the shortest PFS and OS (Figs S6 and S7). In addition, the patients treated in cetuximab arm and with decrease of MAF showed a slightly shorter PFS (7.7 vs 10.1 months, HR 1.62, *P =* 0.212) and OS (15.0 vs 16.6; HR 1.29, *P =* 0.165) compared to bevacizumab‐treated patients (Fig. S7). Due to the small number of patients in the groups, an underpowerment of the statistical values cannot be ruled out.

### 3.6 Patients remaining *BRAF* V600E wild‐type during treatment

In 12 patients with *BRAF* V600 wild‐type status at baseline, the ctDNA status did not change in 11 patients (92%). One patient receiving bevacizumab showed a *BRAF* V600E mutation in follow‐up samples. This patient achieved stable disease within the observation period. Accordingly, survival was favorable in both treatment arms (Fig. 3C).

## 4 Discussion

Understanding the biology of the cancer is much more complex than assessing the status of one mutation. However, *BRAF* V600E mutation is associated with poor survival in mCRC and due to its predictive importance is an important molecular cornerstone in clinical decision making [35, 37, 38, 39]. FIRE‐4.5 is the first randomized study performed in *BRAF* mutant mCRC to compare targeted therapy with cetuximab versus bevacizumab in addition to triplet chemotherapy [26]. Within a prospective research program, liquid biopsies taken at baseline and during follow‐up treatment were analyzed to investigate their clinical relevance in a setting, where *BRAF* V600E mutation as an entry criterion for the FIRE‐4.5 study had previously been shown based on tissue analyses. From a technical standpoint, liquid biopsy is already well‐established to determine *RAS* or *BRAF* gene mutations and readily can be applied if tumor tissue is not available [40, 41, 42, 43, 44].

In general, a concordance of 80–100% is expected when *BRAF* V600E detection based on liquid biopsy or tissue are compared by literature review [14, 21, 45, 46]. In the present investigation, liquid biopsy performed at baseline detected *BRAF* V600E mutation in only 74% of samples, demonstrating a relevant discordance between tissue‐ and liquid biopsy‐based results. Spatial heterogeneity, including intra‐ and interlesional heterogeneity of clonal tumor composition may be one explanation [47]. In addition, temporal heterogeneity may be an issue when archival tissue is evaluated in the advent of metachronous metastasis.

Another argument is that the release of ctDNA into the circulation may vary according to the site of metastases [48, 49]. In this study, the concordance of liquid biopsy and tissue‐based analyses was greatest in the presence of liver metastases (> 92%) but markedly less with peritoneal metastases (60% concordance). This observation is supported by a recently published report from the PEGASUS study evaluating 10 patients experiencing post‐operative relapse with detectable ctDNA in patients with liver metastasis, but not detectable in lung or peritoneal metastases at the point of recurrence [50]. It remains open how the dependence of the ctDNA detectability on the site of metastasis can be caused: mechanisms of ctDNA delivery, the connectivity to the blood system, the number and the location of tumor cells could play a role. This issue must be analyzed in future studies.

In this study, negative testing for *BRAF* V600E mutation in BL was associated with significantly longer PFS as compared to patients with positive tests. In fact, excellent survival times were observed in patients with *BRAF* V600 wild‐type at baseline (PFS 13.2 months; OS 36.7 months) and in contrast, poor outcome data (PFS 6.6 months and OS 13.2 months) were observed in BL *BRAF* V600E mutated patients. The interesting part of this observation is that the results obtained by liquid biopsy really fit to the survival times expected according to tissue‐based mutation status for *BRAF* V600E mutation positive and negative patients [8, 49, 50]. The liquid biopsy analyses can provide a realistic information on the actual mutational status of disease. By consequence, liquid biopsy performed at baseline, before treatment start, turns out to have the potential of an important tool in clinical decision making.

Patients with BL tested positive for *BRAF* mutation showed a clear superiority of FOLFOXIRI plus bevacizumab over FOLFOXIRI plus cetuximab regarding PFS (10.4 months vs 5.7 months; HR 0.4; *P =* 0.009). This finding is well in line with the general conclusion of the FIRE‐4.5 study that bevacizumab‐based chemotherapy is the preferable first‐line treatment of patients with *BRAF* V600E‐mutant mCRC [26].

Absence of *BRAF* V600E mutation in BL on the one hand is associated with rather long survival, on the other hand, prolonged OS is also independent of antibody used in addition to triplet chemotherapy (38.1 months versus 36.7 months). This finding again points out that the biological behavior of this subgroup corresponds to that expected for tissue‐based *RAS*/*BRAF* wild‐type patients [2, 51].

In line with previous publications, the MAF of *BRAF* V600E mutations in ctDNA declines during therapy [35, 36]. It is speculated that clearance from mutant ctDNA might be dependent on bevacizumab treatment [17, 52]. In the current study, each treatment strategy included a targeted agent and the number of patients converted to *BRAF* V600 wild‐type status in liquid biopsy samples was not significantly different between both treatment arms: in CET treatment arm with follow‐up samples 12 of 27 (44%) patients and in the BEV treatment arm 6 of 12 (50%) patients converted (*P =* 0.9). To date, it is unclear whether the release of the cell‐free DNA or the reduction of mutated cell clones in the tumor mass is the reason for MAF reduction in blood [18, 19, 27, 52].

Conversion from *BRAF* V600E mutant status to a *BRAF* V600 wild‐type status was associated with a superior outcome as compared to stable or increased mutational allele frequency. The underlying biology of this observation can be explained on several levels. Complete clearance of *BRAF* V600E mutant cell‐free DNA from circulation may reflect the elimination of this subclone and thus points to excellent susceptibility to treatment [19, 53, 54, 55]. It may, however, also indicate the reduction of this clone to a sub‐detection level, and finally may also relate to elimination of certain metastatic sites known to shed more tumor DNA than others. The clinical relevance of a “neo‐wild‐type” status with regard to targeted therapy is under discussion.

An increase in mutational load was exclusively observed in patients receiving FOLFOXIRI plus cetuximab. This finding refers to the selective pressure induced by cetuximab, but apparently not by bevacizumab. Follow‐up evaluation of liquid biopsies may thus be indicated in patients receiving anti‐EGFR therapy, but not during anti‐VEGF treatment.

While FIRE‐4.5 is the first randomized study published in the subgroup of *BRAF* V600E mutant mCRC, the small number of patients with evaluable liquid biopsies is a clear limitation of this prospective investigation. Despite the prospective nature of this research, observations are therefore essentially hypothesis generating and require verification in further studies.

## 5 Conclusion

In conclusion, the present investigation underlines the power of liquid biopsy as a clinical tool to guide first‐line therapy in mCRC and also illustrates the impact of metastatic patterning to ctDNA release at advanced stages. Detection of cell‐free circulating tumor DNA is not only prognostic, but also predictive regarding the use of antibody‐combined chemotherapy. Liquid biopsy can be used with a short turn‐over time thus allowing a real‐time approach including follow‐up analyses at low risk for the patient.

## Conflict of interest

SKS, WS, TM, RS, SH declare no conflicts of interest. *Alexander Baraniskin*: Honoraria: Merck Serono, Amgen, Sysmex Inostics; Consulting or Advisory Role: Sysmex Inostics; Research Funding: Merck Serono (Inst), Sysmex Inostics (Inst). *Kathrin Heinrich*: Honoraria: Roche Pharma AG, Taiho Pharmaceutical; Consulting or Advisory Role: Servier (Inst); Travel, Accommodations, Expenses: Amgen, Lilly, Servier, Merck KGaA. *David Tougeron*: Honoraria: Amgen, Roche, Sanofi, Bristol Myers Squibb, Merck Serono, MSD, Bristol Myers Squibb, Servier/Pfizer, Ipsen, Pierre Fabre, AstraZeneca, Takeda, BeiGene; Consulting or Advisory Role: Sanofi, MSD, Pierre Fabre, AstraZeneca, Novartis, Takeda; Research Funding: AstraZeneca (Inst), Servier (Inst), Roche (Inst), MSD (Inst), BTG (Inst); Travel, Accommodations, Expenses: Roche, Amgen, Bristol Myers Squibb, MSD, Pierre Fabre. *Dominik Paul Modest*: Honoraria: Merck Serono, Amgen, Servier, Bristol Myers Squibb, Taiho Pharmaceutical, Merck Sharp & Dohme, Pierre Fabre, Onkowissen, Sanofi, Lilly, AstraZeneca/MedImmune, Incyte, Takeda; Consulting or Advisory Role: Merck Serono, Amgen, Merck Sharp & Dohme, Roche, Servier, Incyte, Bristol Myers Squibb, Pierre Fabre, Lilly, Cor2Ed, IQvia, Onkowissen; Research Funding: Amgen (Inst), Servier (Inst); Travel, Accommodations, Expenses: Amgen, Merck Serono, Servier. *Ingo Schwaner*: Honoraria: AbbVie, Janssen, Amgen; Consulting or Advisory Role: AbbVie, Janssen, Novartis, Roche Pharma; AG, Lilly, BeiGene, AstraZeneca, Servier; Expert Testimony: BeiGene; Travel, Accommodations, Expenses: Janssen, BeiGene, Servier. *Florian Kaiser*: Consulting or Advisory Role: Astellas Pharma, GlaxoSmithKline, MSD, Novartis, Sanofi, Pierre Fabre, Elsevier, Servier; Christoph Kahl; Travel, Accommodations, Expenses: Celgene, Celgene. *Meinolf Karthaus*: Consulting or Advisory Role: Amgen; Travel, Accommodations, Expenses: Amgen. *Christian Müller*: Consulting or Advisory Role: Lilly, Amgen, Bristol Myers Squibb; Germany. *Christof Burkart*: Consulting or Advisory Role: Boehringer Ingelheim, Bayer, Amgen; Consulting or Advisory Role: Roche/Genentech, Merck Serono, Amgen, Lilly, Bayer, Servier, Pierre Fabre, Incyte, Daiichi Sankyo Europe GmbH, MSD, BMSi, AstraZeneca. *Sebastian Stintzing*: Honoraria: Merck KGaA, Roche, Amgen, Servier, MSD, Pfizer, Pierre Fabre, Bristol Myers Squibb GmbH, Nordic Bioscience, AstraZeneca; Consulting or Advisory Role: Merck KGaA, Roche, Amgen, Pierre Fabre, MSD, AstraZeneca, Servier, GlaxoSmithKline, TERUMO, Nordic Bioscience, Seagen; Research Funding: Pierre Fabre (Inst), Roche Molecular Diagnostics (Inst), Merck Serono (Inst), Amgen (Inst); Travel, Accommodations, Expenses: Merck KGaA, Roche, Sanofi, Bayer, Sirtex Medical, Amgen, Lilly, Takeda, Pierre Fabre, AstraZeneca. *Volker Heinemann*: Honoraria: Roche, Amgen, Sanofi, Merck, Servier, Pfizer, Pierre Fabre, AstraZeneca, MSD, Seagen; Consulting or Advisory Role: Merck, Amgen, Roche, MSD, Bristol Myers Squibb, MSD Oncology, Novartis, Pierre Fabre, TERUMO, GlaxoSmithKline, Servier/Pfizer, AstraZeneca, Oncosil, Nordic Bioscience; Research Funding: Merck (Inst), Amgen (Inst), Roche (Inst); Travel, Accommodations, Expenses: Merck; No other potential conflicts of interest were reported.

## Author contributions

SK‐S, AB contributed equally to the work. AB, WS, SK‐S, RS, SS, VH designed the translational project. WS, KH, DT, DPM, IS, JE, RP, MS, FK, CK, MK, CM, CB, AB, TM, RS, SS, and VH recruited the patients, provided and analyzed the data and reviewed the manuscript critically. SK‐S, SH compiled the data and the statistical analyses. SK‐S, SH, AB, SS, VH interpreted the data. SK‐S, AB, TM, VH wrote the main manuscript. VH and SS initiated the phase 2 trial. All authors edited the manuscript and approved the final version of this work.

### Peer review

The peer review history for this article is available at https://www.webofscience.com/api/gateway/wos/peer‐review/10.1002/1878‐0261.13778.

## Supporting information


**Fig. S1.** Liquid biopsy‐based mutant allele frequencies (MAF %) at baseline and their changes during treatment.
**Fig. S2.** Characteristics of the *BRAF* V600E digital droplet PCR assay with the QX200 system.
**Fig. S3.** *BRAF* V600E digital droplet PCR results of baseline liquid biopsy samples.
**Fig. S4.** Baseline sample results considering primary side of tumors and metastatic sites.
**Fig. S5.** Survival curves (Kaplan–Meier) of patients grouped by metastatic sites.
**Fig. S6.** Grouping patients according to changes in MAF% of follow‐up samples and their response rates.
**Fig. S7.** Survival curves of patients with BL *BRAF* V600E mutant status categorized into groups according to treatment arms and changes in MAF% at follow‐up.
**Table S1.** Overview of the liquid biopsy sample collection for the FIRE‐4.5 study.
**Table S2.** Data Summary of patient outcomes.

## Data Availability

The data that support the findings of this study are available in the supplementary material of this article.
