# Supplementary material for: Evaluation of circulating tumor DNA as a prognostic and predictive biomarker in BRAF V600E mutated colorectal cancer—results from the FIRE‐4.5 study
Source: Mol Oncol. 2024 Dec 4;19(2):344–56. doi: 10.1002/1878-0261.13778 (PMC11793001; doi:10.1002/1878-0261.13778)
Supplement: Supplementary file 1 — Fig. S1. Liquid biopsy‐based mutant allele frequencies (MAF %) at baseline and their changes during treatment. Fig. S2. Characteristics of the BRAF V600E digital droplet PCR assay with the QX200 system. Fig. S3. BRAF V600E digital droplet PCR results of baseline liquid biopsy samples. Fig. S4. Baseline sample results considering primary side of tumors and metastatic sites. Fig. S5. Survival curves (Kaplan–Meier) of patients grouped by metastatic sites. Fig. S6. Grouping patients according to changes in MAF% of follow‐up samples and their response rates. Fig. S7. Survival curves of patients with BL BRAF V600E mutant status categorized into groups according to treatment arms and changes in MAF% at follow‐up. Table S1. Overview of the liquid biopsy sample collection for the FIRE‐4.5 study. Table S2. Data Summary of patient outcomes. [file MOL2-19-344-s001.pdf]

Supporting information to „Evaluation of circulating tumor DNA as a prognostic and predictive biomarker in *BRAF* V600E mutated colorectal cancer – results from FIRE-4.5 Study" Klein-Scory et al.

Supplementary Table 1 Overview of the liquid biopsy sample collection for the FIRE-4.5 study

					Baseline liquid biopsy samples (BL) Pre-therapy		Follow up samples		
**#samples LB per patient**	#Pats	analysis	LB samples before or at first Dose	with Follow Up	BL-1	BL0	FU1	FU2	FU3
				Median (days)	-3	0	60	175	314.5
				Interquartile range (days)	-6.5 to -1.5	0	56-68.5	132-211	275-420
**1**	10	excluded	no	yes			7	3	
	**15**	**included**	**yes**	**no**	**5**	**10**			
**2**	4	excluded	no	yes			3	3	2
	**28**	**included**	**yes**	**yes**	**10**	**18**	**21**	**6**	**1**
**3**	**20**	**included**	**yes**	**yes**	**7**	**13**	**17**	**18**	**5**
**4**	**3**	**included**	**yes**	**yes**		**3**	**3**	**3**	**2**
	80		**66 patients (143 samples)**	**51 patients (128 samples)**	23	44	51	33	10
					All samples 161				

Liquid biopsy samples (LB) were taken at baseline (pre-therapy) and at predefined intervals within the first 2 months thereafter including the timepoint of first progression. Following an approach of quality assurance, testing was performed in a central laboratory and highly sensitive methodology was applied using the OncoBEAM CRC IVD test for *RAS* mutation and droplet PCR for *BRAF* V600E mutation. This approach contrasts with the routine procedure for tissue testing, where *RAS*- and *BRAF* analyses are carried out decentral and with different test methods.

Supplementary Table 2 Data summary of patient outcomes

Baseline samples			PFS	OS	DCR	ORR		CET = FOLFOXIRI plus cetuximab						BEV = FOLFOXIRI plus bevacizumab					
		N	months	months	%	%	**NE***	N (%)	PFS	OS	DCR	ORR	**NE***	N (%)	PFS	OS	DCR	ORR	**NE***
**all**	median	80	7	**14.5**	83	54	10	56 (70)	**6.9**	**13.3**	80	49	7	24 (30)	**9.6**	**17**	90.5	67	3
	range		0.03-21.9	0.75-50.4					0.03-19.2	0.75-36.8					0.03-21.9	2.4-32.8			
**with BL**	median	66	6.5	15.9	81	56	7	47(71)	6.6	**13.2**	79	50	5	19 (29)	**10.6**	**22.7**	88	71	2
	range		0.03-19.1	0.75-36.8					0.03-18.4	0.75-36.8					0.03-19.1	2.7-32.8			
**BL mut *BRAF V600E***	median	49	6.6	**13.2**	77	55	5	36 (73)	**5.7**	**11.6**	72	44	4	13 (27)	**10.4**	**16.6**	92	83	1
	range		0.2-19	0.7-41.9					0.2-18.4	2.4-41.9					1.6-19	0.7-41.6			
**BL wt *BRAF* V600**	median	17	13.2	**36.8**	93	60	2	11 (65)	**11.3**	**38.1**	100	70	1	6 (35)	**17**	**36.7**	80	40	1
	range		0.03-17.3	2.4-50.4					0.03- 17.3	2.4-50.4					0.03-17	2.9-41.9			

**Follow-up samples**			**PFS**	**OS**	**DCR**	**ORR**		**CET = FOLFOXIRI plus cetuximab**						**BEV = FOLFOXIRI plus bevacizumab**					
		N	months	months	%	%	**NE***	N (%)	PFS	OS	DCR	ORR	**NE***	N (%)	PFS	OS	DCR	ORR	**NE***
**BL mut FU remained mut**	median	21	3.9	10.6	63	42	2	15 (76)	3.7	9.8	57	29	1	6 (24)	8.3	13.1	80	80	1
	range		0.03-18.4	2.1-43.1					0.03-18.5	2.4-39.7					1.9-10.7	4.0-17.0			
**BL mut FU converted to BRAF wt**	median	18	8.6	17.4	89	67	0	12 (66)	7.8	12.1	83	58	0	6 (34)	11.8	22.7	100	83	0
	range		0.9-18.9	6.7-41.9					0.8-12.8	5.3-36.5					7.45-19	12.6-32.8			
**BL mut FU Increased or stable MAF**	median	6	2.3	5.1	20	20	1	6 (100)	2.3	5.1	20	20	1	0					
	range		0.03-2.3	1.5-8.4					0.03-4.1	2.1-8.4									
**BL mut FU decreased MAF**	median	33	7.9	16.6	81	59	1	21 (66)	7.7	15.0	76	48	0	12 (34)	10.1	16.6	91	80	1
	range		0.94-18.9	2.5-43.1					0.9-18.4	2.5-43.3					1.9-22.0	4-41.6			
**BL wt with FU remained wt**	median	11	17.0	36.8	91	55	0	8 (67)	13.3	38.1	100	63	0	3 (33)	17.0	36.7	67	33	0
	range		13.8-20.8	11.9-50.4					4.3-20.7	16.6-50.4					1.8-25.9	11.9-39.3			
**BL wt with FU changed to mut**	median	1	0.03	7.9	100	0	0						0	1	0.03	36.7	100	0	0

Compilation of data to the progression-free survival (PFS) and overall survival (OS) calculated by Kaplan-Meier estimation, as well as the data to disease control rate (DCR) and overall response rate (ORR). Grouping of patients by categorisation of the mutational allele frequencies of *BRAF* V600E. BL, baseline Liquid biopsy sample, FU Follow-up samples after onset of therapy. *NE Number of patients non evaluated in respect to the responses at data cut-off 01/2022

Supplementary Figure 1

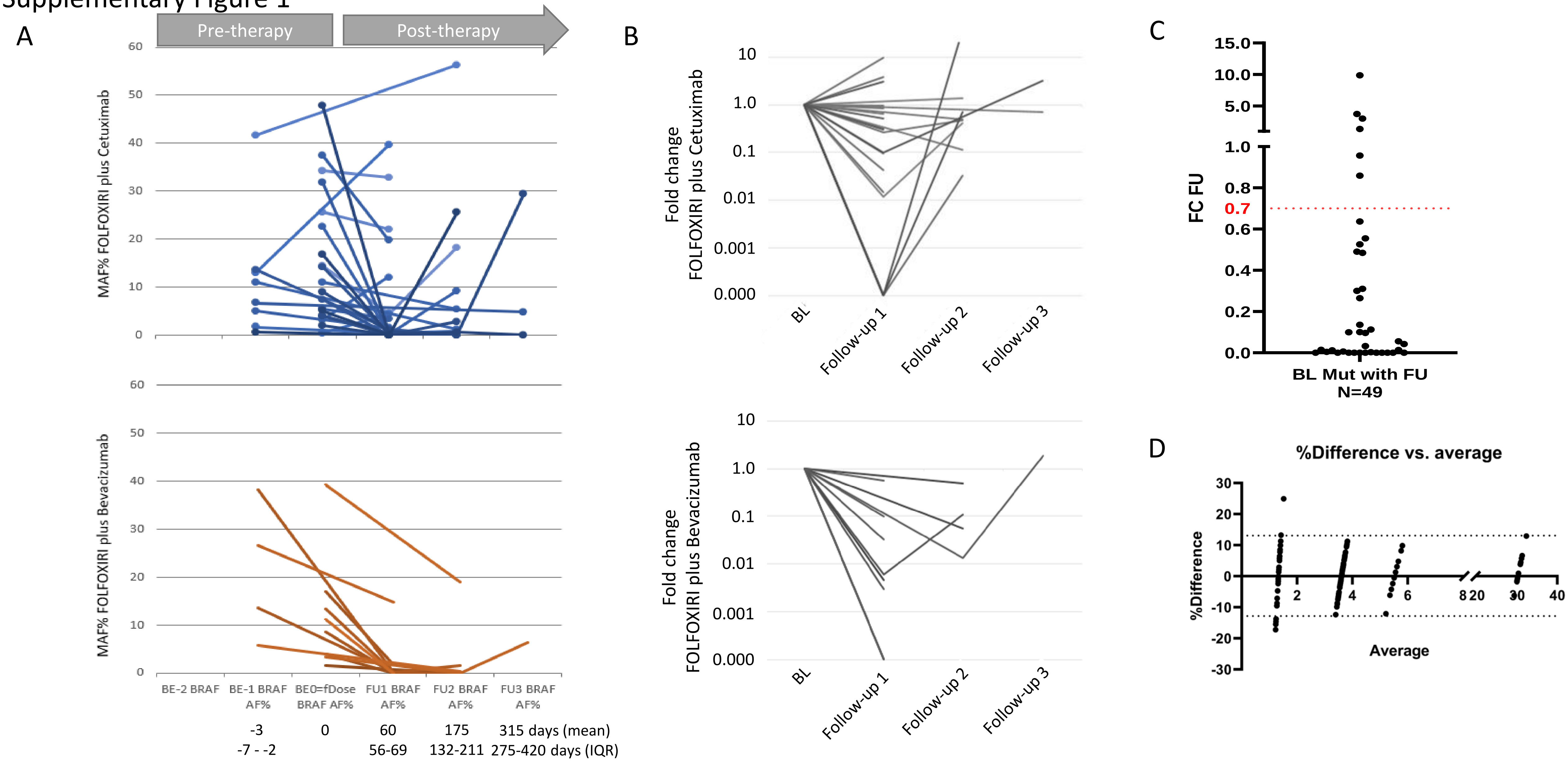

Supplementary Figure 1 Liquid biopsy based mutant allel frequency (MAF %) at baseline and its changes during treatment. A Dynamic change of MAF % during treatment: blue, treated FOLFOXIRI plus Cetuximab; red, treated with FOLFOXIRI plus Bevacizumab. IQR= Interquartile range **B** Fold change of MAF % altered during treatment in relation to baseline value calculated by AF% follow-up (first available) / AF% baseline. C The fold change (FC) of MAF% of follow-up samples versus baseline. **D** A MAF % deviation of more than 30% of the MAF baseline value was chosen to ensure that ouliers were not considered relevant changes.

Supplement Figure 2

A

		Referenc e value	Mean	Standard Deviation	Coefficient of variation	CI 90%	n
NTC	MAF%	0	0	0	0.00	0.000	125
NC	MAF% 0	0	0.004	0.02	5.77	0.004	105
NC HD249	events	0	0.5	0.71	1.41	3.16	4
NC HD249	MAF%	0	0.005	0.007	1.41	0.03	4
PC HT29	MAF%	30	31.4	1.71	0.05	1.06	9
PC HD701	MAF%	10.5	9.93	0.66	0.07	2.94	4
PC1	MAF% 6	6	5.52	0.34	0.06	0.14	21
PC2	MAF% 3.5	3.5	3.63	0.27	0.07	0.04	87
PC3	MAF% 3	3	3.11	0.31	0.10	0.09	33
PC4	MAF% 1.5	1.5	1.33	0.14	0.11	0.04	36

B

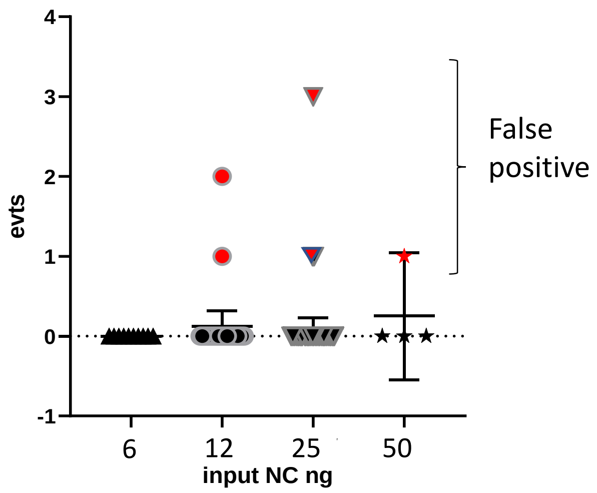

C

Cut-off	Sensitivity%	95% CI	Specificity%	95% CI	Likelihood ratio
> 0.1357	100.0	90.36% to 100.0%	99.05	94.80% to 99.95%	105.0

Standard deviation of NC N=105:	0.02
**Real world cut-off:**	**0.17**

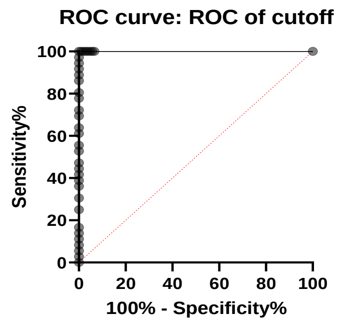

		Sensitivity%	95% CI	Specificity%	95% CI	Likelihood ratio
1	> 0.004557	100.0	90.36% to 100.0%	93.33	86.87% to 96.73%	15.00
2	> 0.01275	100.0	90.36% to 100.0%	94.29	88.09% to 97.36%	17.50
3	> 0.01748	100.0	90.36% to 100.0%	95.24	89.33% to 97.95%	21.00
4	> 0.02703	100.0	90.36% to 100.0%	96.19	90.61% to 98.51%	26.25
5	> 0.04452	100.0	90.36% to 100.0%	97.14	91.93% to 99.22%	35.00
6	> 0.05696	100.0	90.36% to 100.0%	98.10	93.32% to 99.66%	52.50
7	> 0.1357	100.0	90.36% to 100.0%	99.05	94.80% to 99.95%	105.0
8	> 0.6405	100.0	90.36% to 100.0%	100.0	96.47% to 100.0%	
9	> 1.075	97.22	85.83% to 99.86%	100.0	96.47% to 100.0%	

D

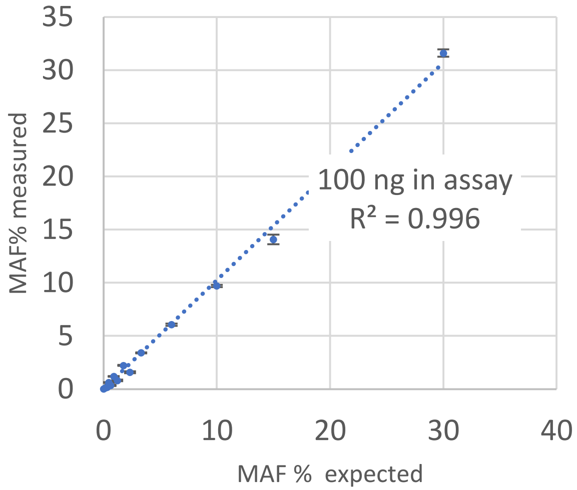

E

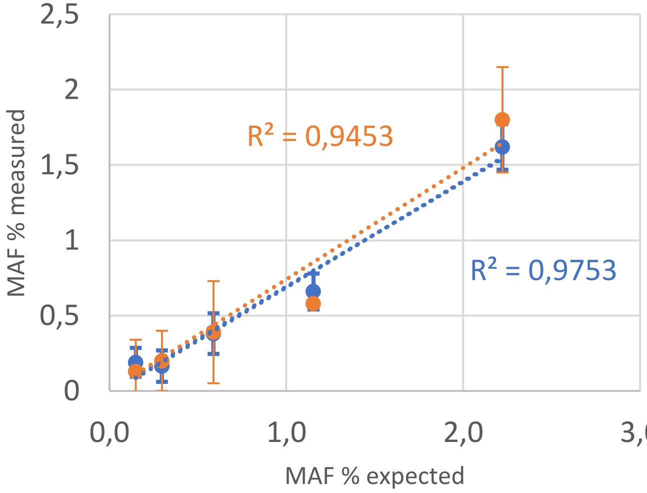

F

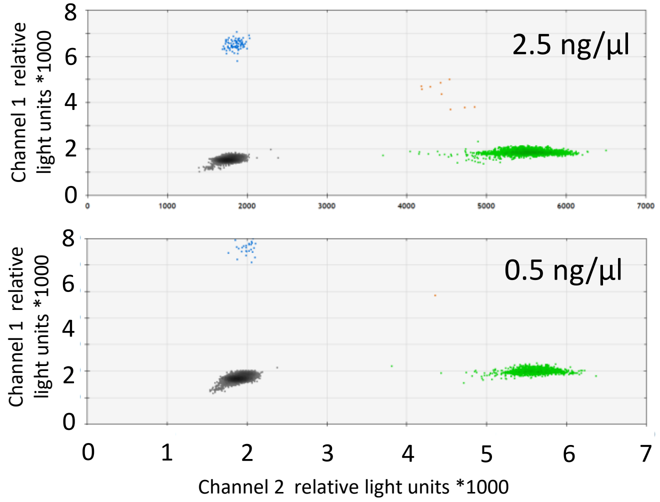

Supplementary figure 2 Characteristics of the *BRAF* V600E digital droplet PCR assay with QX200 system. A Compilation of mutational allele frequency (MAF %) values of the *BRAF* V600E ddPCR assay with reference materials and „Spike In" controls with genomic DNA of cell lines (PC1-PC4). NC, negative control; NTC, non template control **B** The false detection rate increased with input amount of DNA to the assay. Considering the false positive events (evts) detected in negative samples, the cut-off limit of events was set to 3 events. **C** ROC analysis gave a MAF% Cut-off of 0.136 had a sensitivity of 100 % and a specificity of 99.05 %. Including the standard deviation of false positive results, the real word cut-off was set to 0.17. **D** Linearity of *BRAF* V600E assay starting with 30% MAF with 100 ng whole amount of DNA in assay. **E** The linearity of *BRAF* MUT detection was limited at 0.16 % MAF with the minimal input DNA of 0.5 ng/µl eluate. F Examples of 2D Plot of the assay measured in QX200 system using low input DNA amount.

Supplementary Figure 3

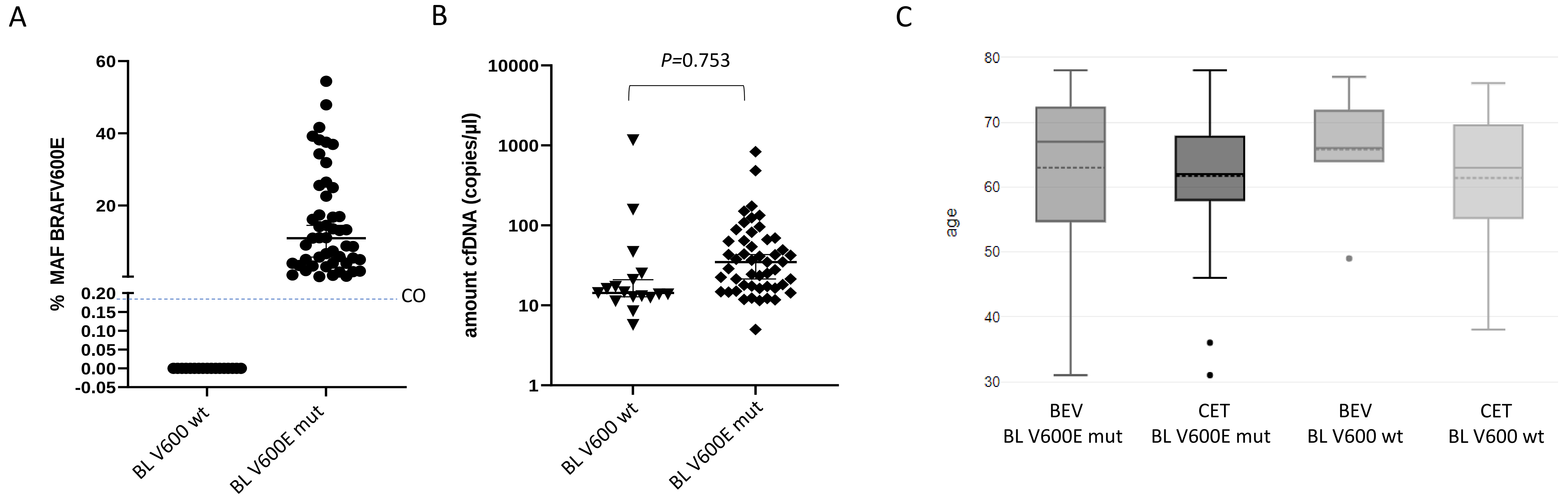

Supplementary figure 3 *BRAF* V600E digital droplet PCR results of baseline liquid biopsy samples. A In the baseline samples from 17 Patients no *BRAF* V600E mutations (no mut events) were detectable, and 49 patients have concordant measurable *BRAF* V600E mutations in the baseline samples. CO = cut-off was set to 0.17 (see supplementary figure 1). **B** The whole amounts of circulating DNA were not different between the baseline samples with or without detectable BRAF mutations (P=0.753; Due to the outliers in the groups, we selected the T-test with Welch´s correction). **C** The age of patients in BL wild-type and mutant group was not different.

Supplementary Figure 4

A

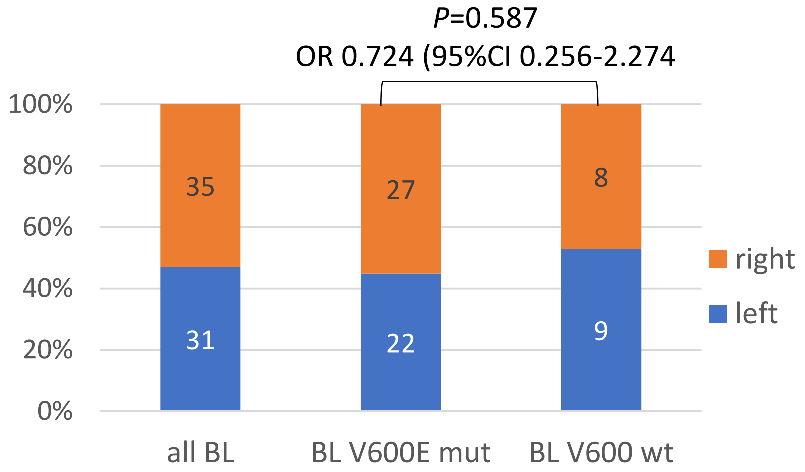

B

metastasis localization		with LB N (%)	BL *BRAF* V600E mut	BL *BRAF* V600 wt	Fisher´s exact	concordance to tissue-based *BRAF* results	PFS Median (months)	P-value (log-rank)	HR 95% CI	OS Median (months)	P-value (log-rank)	HR 95% CI
Multiple including liver	yes	41 (62%)	38	3	OR 16.12 (95%CI 3.795-57.11)	93%	7.5	0.447	1.236	13.3	0.156	1.59
	no		11	14	P<0.0001		7.6		0.72-2.13	20.9		0.84-2.91
liver only*	yes	16 (25%)	14	2	OR 15.87 (95%CI 3.14-73.81)	86%	5.9	0.431	0.764	11.7	0.545	1.250
	no		15	34	P<0.0001		7.7		0.41-1.40	17.0		0.581-2.68
lymph nodes	yes	24 (36%)	22	2	OR 6.11 (95%CI 1.281-28.61)	92%	6.5	0.065	1.64	15.0	0.840	1.64
	no		27	15	P=0.019		7.9		0.91-2.94	16.6		0.91-2.94
peritoneal	yes	23 (35%)	13	10	OR 0.253 (95%CI 0.081-0.827)	57%	7	0.962	0.986	20.9	0.986	0.986
	no		36	7	P=0.021		7.4		0.56-1.72	15.3		0.56-1.72
lung	yes	16 (25%)	10	6	OR 0.470 (95%CI 0.153-1.766)	63%	6.6	0.651	0.855	15.0	0.882	1.057
	no		39	11	P=0.324		7.6		0.44-1.65	16.2		0.49-2.25

Supplementary figure 4 Baseline sample results considering primary side of tumors and metastatic sites
A The proportion of patients with right or left sided tumors was not different in both groups**.** *P*-value calculated by Fisher´s exact test **B** Number of patients considering the main localization sites of metastases. All patients are in advanced stage with metastatic sites. The V600E mutant baseline samples (BL) derived from patients with multimetastatic sites involving liver and with metastatic lymph nodes, while the V600 wild-type samples were more frequently found in samples of patients with peritoneal metastases (peri).
*The annotation about metastasis site in respect to “liver only” was not known for one patient.

Supplementary Figure 5

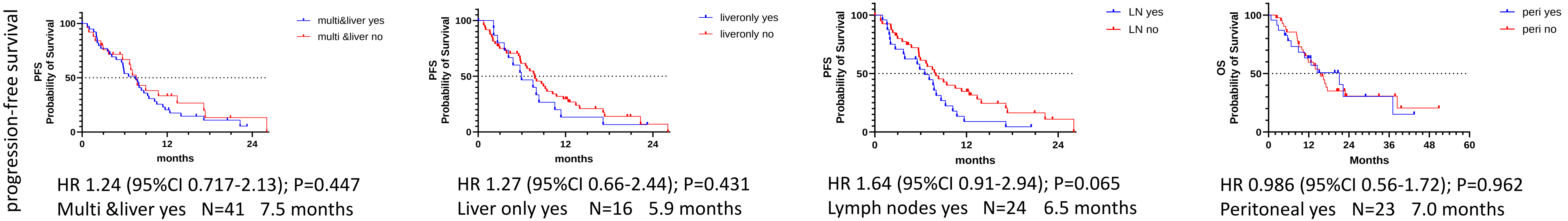

HR 1.24 (95%CI 0.717-2.13); P=0.447
Multi &liver yes N=41 7.5 months
Multi &liver no N=25 7.6 months

HR 1.27 (95%CI 0.66-2.44); P=0.431
Liver only yes N=16 5.9 months
Liver only no N=49 7.7 months

HR 1.64 (95%CI 0.91-2.94); P=0.065
Lymph nodes yes N=24 6.5 months
Lymph nodes no N=42 7.9 months

HR 0.986 (95%CI 0.56-1.72); P=0.962
Peritoneal yes N=23 7.0 months
Peritoneal no N=43 7.4 months

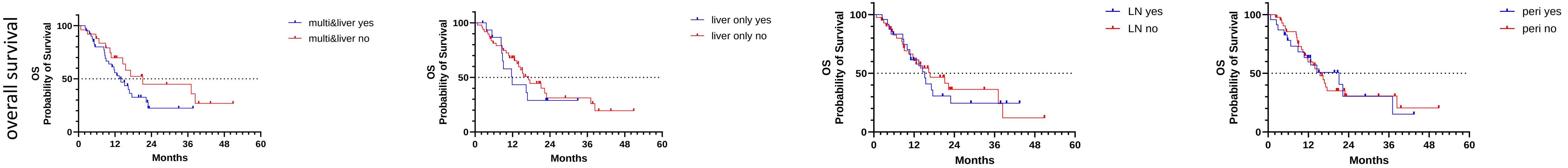

HR 1.59 (95%CI 0.84-2.91); P=0.156
Multi &liver yes N=41 13.3 months
Multi &liver no N=25 20.9 months

HR 1.25 (95%CI 0.581-2.68); P=0.540
Liver only yes N=16 11.7 months
Liver only no N=49 17.0 months

HR 1.64 (95%CI 0.91-2.94); P=0.840
Lymph nodes yes N=24 15.0 months
Lymph nodes no N=42 16.6 months

HR 0.986 (95%CI 0.56-1.72); P=0.962
Peritoneal yes N=23 20.9 months
Peritoneal no N=43 15.3 months

Supplementary Figure 5 Survival curves (Kaplan-Meier) of patients grouped by metastatic sites.
The patients grouped by site of metastases yielded similar PFS (progression-free survival) and OS (overall survival) for these groups. Multi&liver, mutlimetastatic sites including liver; peri, peritoneal metastases; LN, metastatic Lymph node; P-value calculated by log-rank test

Supplementary Figure 6

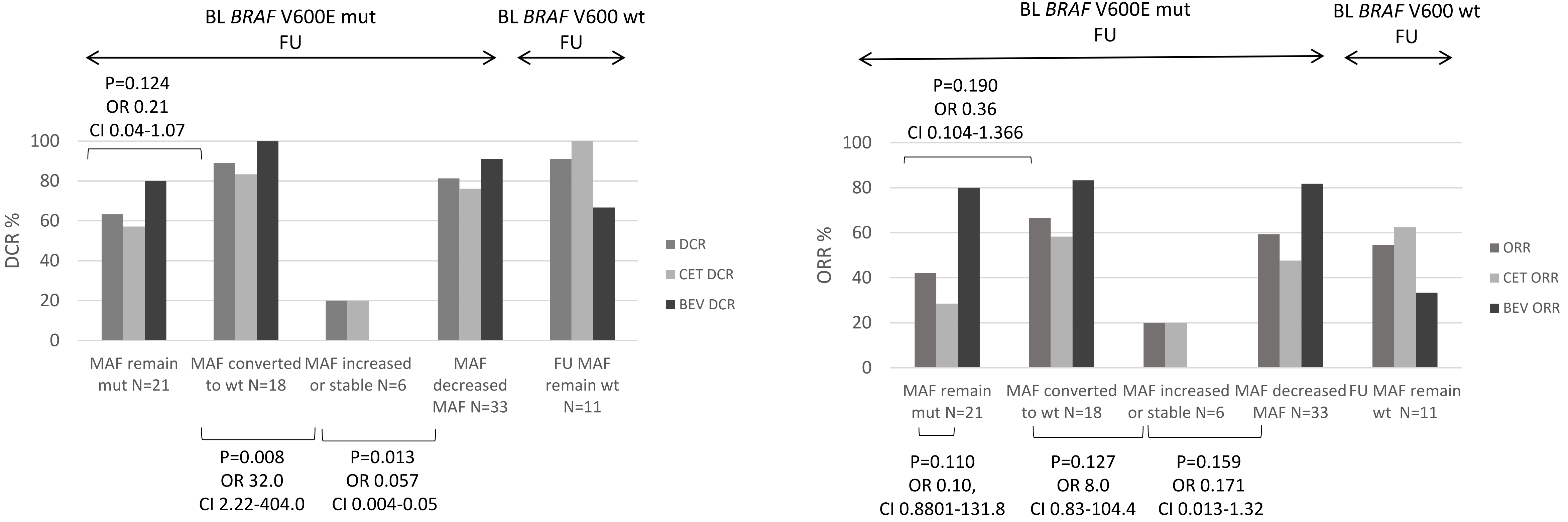

Supplementary figure 6 Grouping patients according to changes in MAF% of follow-up samples and their response rates. The comparisons of disease control rates (DCR %) revealed significant better outcome of patients with decreased mutational allele frequencies (MAF) during treatment than those with increased or remained mutational status. Best DCR reached the group „converted to V600 wt“ and the group of „BL V600 wt“ treated with CET or BEV. BL V600E mutant group had the better ORR (overall response rate) after BEV treatment. CET treatment was only superior to BEV treatment in the group of BL V600 wt group. MAF, mutational allele frequency; CI, 95% confidence interval; Odds ratio (OR) and P-value were calculated by Fisher´s exact test

Supplementary Figure 7

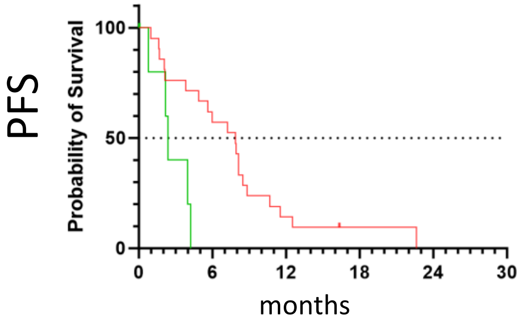

HR 0.294 (95%CI 0.006-1.39); P=0.006
CET MAF decreased N=21 7.7 months
CET MAF increased N=6 2.3 months

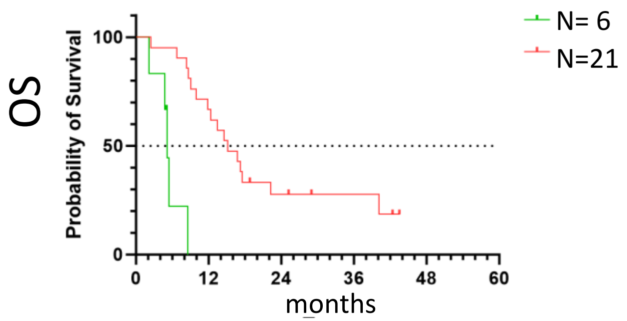

HR 0.158 (95%CI 0.021-1.19); P<0.0001
CET MAF decreased N=21 15.0 months
CET MAF increased N=6 5.1 months

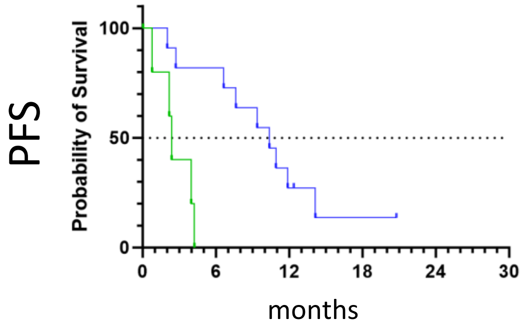

HR 0.223 (95%CI 0.041-1.188); P=0.0014
BEV MAF decreased N=12 10.1 months
CET MAF increased N=6 2.3 months

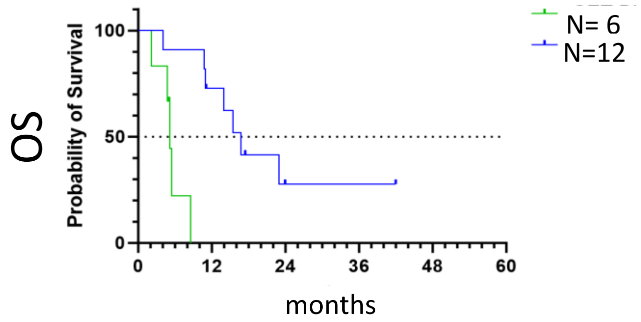

HR 0.198 (95%CI 0.035-1.10); P=0.0007
BEV MAF decreased N=12 16.6 months
CET MAF increased N=6 5.1 months

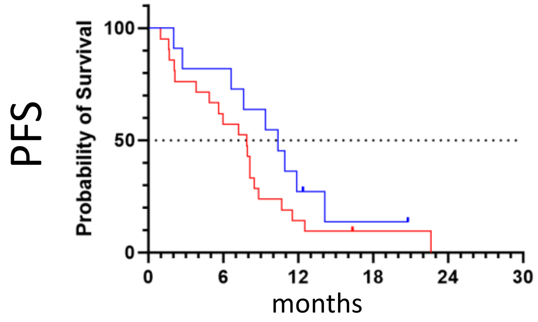

HR 1.62 (95%CI 0.76-3.4); P=0.212
CET MAF decreased N=21 7.7 months
BEV MAF decreased N=12 10.1 months

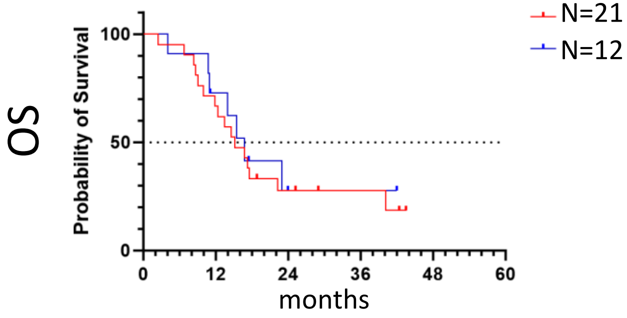

HR 1.20 (95%CI 0.051-2.83); P=0.165
CET MAF decreased N=21 15.0 months
BEV MAF decreased N=12 16.6 months

Supplementary Figure 7 Survival curves of patients with BL *BRAF* V600E mutant status categorised into groups according to treatment arms and changes in MAF% at follow-up. The patients with increased MAF and CET treatment had the worst PFS and OS. On the other hand, some patients in both treatment arms showed reduction in MAF. BEV treatment seems to be slightly beneficial.
